# Prevalence and Characterization of Plasmid-Mediated Quinolone Resistance Determinants *qnr* and *aac(6’)-Ib-cr* in Ciprofloxacin-Resistant *Escherichia coli* Isolates from Commercial Layer in Korea

**DOI:** 10.4014/jmb.2003.03058

**Published:** 2020-05-15

**Authors:** Kwang Won Seo, Young Ju Lee

**Affiliations:** 1 College of Veterinary Medicine and Zoonoses Research Institute, Kyungpook National University, Daegu 41566, Republic of Korea; 2 Department of Basic Sciences, College of Veterinary Medicine, Mississippi State University, MS 39762, USA

**Keywords:** Antimicrobial resistance, commercial layer chicken, *Escherichia coli*, fluoroquinolones, plasmid- mediated quinolone resistance

## Abstract

The prevalence and characterization of plasmid-mediated quinolone resistance (PMQR) determinants in ciprofloxacin-resistant *Escherichia coli* isolated from a Korean commercial layer farm were studied. A total of 45 ciprofloxacin-resistant *E. coli* isolates were recovered and all isolates were multidrug-resistant. Eight isolates have the PMQR genes *aac(6’)-Ib-cr*, *qnrS1,* and *qnrB4,* and seven isolates exhibited double amino acid exchange at both *gyr*A and *par*C, and have high fluoroquinolone minimum inhibitory concentrations. Five transconjugants demonstrated transferability of PMQR and β-lactamase genes and similar antimicrobial resistance. Because PMQR genes in isolates from commercial layer chickens could enter the food supply and directly affect humans, control of ciprofloxacin resistance is needed.

Ciprofloxacin (CIP) is an important synthetic antimicrobial agent for treating bacterial infections in both humans and animals [1]. CIP resistance develops when bacteria alter their response(s) to the use of this antimicrobial drug [2]. Recently, a steady increase in the prevalence of CIP-resistant *Escherichia coli* has been reported worldwide [3, 4]. Along with the recent increase in fluoroquinolone resistance, the prevalence of plasmid-mediated quinolone resistance (PMQR) genes has also been increasing in various Enterobacteriaceae worldwide [5]. Although PMQR determinants confer a low level of quinolone resistance on their own, they also facilitate the acquisition of high-level resistance and resistant mutations among susceptible strains [6].

In Korea, the presence of PMQR genes in *E. coli* isolates from broiler poultry or chicken meat has been reported; however, little is known about PMQR genes in *E. coli* from commercial layer farms, which play an important role in the supply of dietary protein through the production of eggs [7]. Unlike broiler chickens, commercial layer hens are raised in the cage system past 70 weeks of age [8]. Their eggs can be contaminated by penetration through the eggshell from the colonized gut, or from contaminated feces during or after oviposition, and by infections originating from the reproductive organs [9, 10]. Especially, contaminated eggs are a common cause of food poisoning in humans and can cause serious illnesses like *Salmonella* infection [11]. In this study, we surveyed genetically characterized CIP-resistant *E. coli* isolates from commercial layer farms in Korea.

Forty-five *E. coli* isolates demonstrating CIP resistance were selected from among 320 *E. coli* samples collected from feces (from chickens 20 weeks of age) and dust in 16 commercial layer farms (62 flocks) during the period between 2015 and 2017. Using the diffusion test, all CIP-resistant *E. coli* isolates were investigated for antimicrobial resistance using drugs from 9 classes applied to 18 antimicrobial discs (BD Biosciences, Sparks, MD, USA). MIC ranging from 0.06 to 512 mg/L to NAL, CIP, and enrofloxacin (ENR) were determined using standard agar dilution methods according to recommendations of the Clinical & Laboratory Standards Institute [12]. Multidrug-resistance (MDR) was defined as acquired non-susceptibility to at least 1 agent in 3 or more antimicrobial categories [13].

Polymerase chain reaction amplification of the PMQR markers *qnrA*, *qnrB*, *qnrC*, *qnrD*, *qnrS,* and *aac(6’)-Ib-cr*, and b-lactamase genes *bla*_CTX-M_, *bla*_TEM_, *bla*_SHV_, and *bla*_OXA_, was performed as previously described [14-16]. The transfer of PMQR genes and b-lactamase genes was performed by conjugation experiments using the broth mating method with sodium azide-resistant *E. coli* J53 as a recipient [17]. Transconjugants were selected on MacConkey agar (BD Biosciences) plates with sodium azide (100 μg/ml; Sigma-Aldrich, USA) and ampicillin or tetracycline (100 μg/ml; Sigma-Aldrich). To identify mutations in the quinolone-resistance determining region, PCR was performed as specified by Rodríguez-Martínez *et al.* (2006) and Vasilaki *et al.* (2008), respectively [18, 19].

Among the 45 CIP-resistant *E. coli* isolates from commercial layer farms, all isolates showed MDR against 3 to 9 classes of antimicrobial agents (Table 1). The resistance rate against 6 and 7 classes was 20.0% and 31.1%, respectively. Although 100% of the isolates showed resistance to quinolones, > 70% of the isolates showed resistance to penicillins, tetracyclines, and aminoglycosides. However, only 6.7% of isolates showed resistance to β-lactam/β-lactamase inhibitor combinations. The distributions of the 8 PMQR-positive *E. coli* among the 45 CIP resistant-*E. coli* isolates are shown in Table 2. Two *qnr* genes (*qnrS1* and *qnrB4)* were identified in 5 and 1 *E. coli* isolate(s), respectively, while *aac(6’)-Ib-cr* genes were detected in 2 *E. coli* isolates. Among the 8 PMQR-positive *E. coli* isolates, 2 b-lactamase genes (*bla*_CTX-M-15_ and *bla*_TEM-1_) were identified in 2 and 3 *E. coli* isolates, respectively. In the conjugation test, 5 transconjugants demonstrated transferability of PMQR genes and b-lactamase genes and similar antimicrobial resistance (Table 2).

Seven PMQR-positive isolates exhibited double amino acid exchange at both *gyr*A and *par*C, and MICs ≥ 16 mg/l of CIP and ENR were observed against these isolates. Furthermore, one PMQR-positive isolate exhibited a single amino acid exchange at *par*C, and MIC ≥ 4 mg/l and ≥ 8 mg/l of CIP and ENR were observed against this isolate, respectively (Table 2).

In this study, 100% of CIP-resistant *E. coli* isolates showed MDR against 3 to 9 classes of antimicrobial agents. In a previous study, Wasyl *et al.* (2013) reported that 31.0% of MDR *E. coli* was identified from commercial layer farms in the European Union [3]. The difference in MDR between the commercial layer farms in Korea and those of other countries may also be associated with the use of antibiotics in each country. Aalipour *et al.* (2014) reported that antibiotic consumption (per 1 kg of animal products) in the European Union and United States is 21 and 94 mg/kg/year, respectively, while that in Korea has been reported to be 285.7 mg/kg/year [20]. Especially, in Korea, the mass medication of poultry with fluoroquinolones is still permitted, and the sale volume of enrofloxacin is the highest among all antimicrobials used to treat poultry [21]. In addition, the Korea Animal and Plant Quarantine Agency reported that *E. coli* with resistance to CIP in poultry has increased from 37.0% in 2007 to 80.0% in 2015 [22].

Many studies have reported that the prevalence of PMQR genes varies from 2.2% to 57.0% globally, depending on the resistance mechanisms of the animal [23, 24]. In this study, the *qnrB4*, *qnrS1,* and *aac(6’)-Ib* genes were identified in 2.2% (1/45), 11.1% (5/45), and 2.2% (1/45) of CIP resistant-*E. coli* isolates, respectively, from a commercial layer farm. The frequency of *qnrS1* and *aac(6’)-Ib* was significantly higher than that in other animal studies [*qnrS1* (2.2%) and *aac(6’)-Ib* (1.1%)] in Korea, although the frequency of *qnrB4* (0.5%) was low [25]. To establish the association between mutation and the presence of these PMQR determinants, *gyr*A and *par*C were sequenced in PMQR-positive isolates. Among 8 PMQR-positive *E. coli* isolates, 7 exhibited a double amino acid exchange in both *gyr*A and *par*C, and MICs ≥ 16 mg/l of CIP and ENR were observed against these isolates. In this study, the transconjugants expressed similar antimicrobial resistance patterns and revealed the presence of PMQR genes (*qnrS1*, *qnrB4*, and *aac(6’)-Ib-cr*) and b-lactamase genes (*bla*_CTX-M-15_ and *bla*_TEM-1_). This result is also consistent in that these transconjugants have similar antimicrobial resistance patterns and the same antimicrobial resistance genes of the donor strains in the previous study [26]. Therefore, this result suggests wide dissemination of PMQR and b-lactamase genes through plasmids via horizontal transfer, and that poultry can contribute to the transmission of these genes to humans.

To our knowledge, this study was the first to investigate the molecular characteristics of CIP-resistant *E. coli* isolates from a commercial layer farm in Korea. We demonstrated that PMQR genes have a relatively high prevalence (17.8%) in CIP-resistant *E. coli* isolates with related susceptibility to fluoroquinolones, revealing that *qnrS, qnrB,* and *aac(6’)-Ib* are the most prevalent PMQR genes in *E. coli* isolates from a commercial layer farm. Additionally, our findings suggest that there is a need to emphasize and enforce rational use of antimicrobials, and that regular antimicrobial susceptibility surveillance is essential in commercial layer chicken farming.

## Figures and Tables

**Table 1:** Distribution of multidrug-resistance patterns among 45 ciprofloxacin-resistant *E. coli* isolates from commercial layer.

Antimicrobial resistance class pattern^a^	Frequency	Prevalence (%)
**Nine of classes**	**2**	**4.4**
AMGs, BL/BLICs, CEPs, FPIs, FQs, PCNs, PHs, Qs, TETs	2	4.4
**Eight of classes**	**4**	**8.9**
AMGs, CEPs, FPIs, FQs, PCNs, PHs, Qs, TETs	4	8.9
**Seven of classes**	**14**	**31.1**
AMGs, CEPs, FPIs, FQs, PCNs, Qs, TETs	5	11.1
AMGs, FPIs, FQs, PCNs, PHs, Qs, TETs	4	8.9
AMGs, CEPs, FPIs, FQs, PCNs, Qs, TETs	2	4.4
AMGs, CEPs, FQs, PCNs, PHs, Qs, TETs	1	2.2
AMGs, CEPs, PCNs, PHs, Qs, FPIs	1	2.2
CEPs, FPIs, FQs, PCNs, PHs, Qs, TETs	1	2.2
**Six of classes**	**9**	**20.0**
AMGs, CEPs, FQs, PCNs, Qs, TETs	6	13.3
AMGs, FQs, PCNs, PHs, Qs, TETs	2	4.4
AMGs, FPIs, FQs, PCNs, Qs, TETs	1	2.2
**Five of classes**	**7**	**15.6**
AMGs, FQs, PCNs, Qs, TETs	4	8.9
CEPs, FPIs, FQs, PCNs, Qs	1	2.2
CEPs, FPIs, FQs, Qs, TETs	1	2.2
CEPs, FQs, PHs, Qs, TETs	1	2.2
**Four of classes**	**3**	**6.7**
BL/BLICs, CEPs, FQs, Qs	1	2.2
CEPs, FQs, Qs, TETs	1	2.2
FPIs, FQs, PCNs, Qs	1	2.2
**Three of classes**	**6**	**13.3**
CEPs, FQs, Qs	6	13.3
Total	45	100.0

a AMGs, aminoglycosides; BL/BLICs, β-lactam/β-lactamase inhibitor combinations; CEPs, cephems; F pathway inhibitors; FQs, fluoroquinolones; PCNs, penicillins; PHs, phenicols; Qs, quionolones; TETs, tetracyclines.

**Table 2:** Characteristics of the 8 plasmid-mediated quinolone resistance-positive *E. coli *isolates from commercial layer.

Isolate	Farm	PMQR genes^a^	β-lactamase genes	Antimicrobial resistance pattern^b^	MICs (µg/mL)^c^			QRDR mutations^d^	
	
					NA	ENR	CIP	*gyr*A	*par*C
Donor									
Gi-CC-4	Farm 3	*qnrS1*	CTX-M-15	AM, CZ, CF, CXM, CTX, CAZ, TE, SXT, G, C	≥512	64	16	S83L, D87N	S80I
Gi-CC-5	Farm 3	*qnrS1*	CTX-M-15	AM, AMC, CZ, CF, CXM, CTX, CAZ, TE, SXT, G, C	≥512	128	32	S83L, D87N	S80I
Gi-CC-7	Farm 3	*qnrS1*	-	AM, TE, G	≥512	8	4	WT	S80I
Gi-CC-27	Farm 4	*aac(6’)-Ib-cr*	TEM-1	AM, CTX, TE, SXT, G	≥512	32	16	S83L, D87N	S80I
Gi-CC-28	Farm 4	*aac(6’)-Ib-cr*	TEM-1	AM, CZ, CF, TE, SXT, G	≥512	64	16	S83L, D87N	S80I
Gi-CC-35	Farm 5	*qnrB4*	-	AM, TE, SXT, G, C	≥512	32	32	S83L, D87N	S80I
Gi-CC-36	Farm 5	*qnrS1*	-	AM, CZ, CF, TE, SXT, G	≥512	16	16	D87N	S80I
Gi-CC-37	Farm 5	*qnrS1*	TEM-1	AM, CZ, FOX, TE, G	≥512	32	32	S83L, D87N	S80I
Recipient									
*E. coli* J53		NT	NT	NT	4	0.06	0.06	NT	NT
Transconjugants									
Gi-CC-4-T		*qnrS1*	CTX-M-15	AM, CZ, CXM, CTX, CAZ, TE, SXT, G, C	4	0.125	0.25	NT	NT
Gi-CC-5-T		*qnrS1*	CTX-M-15	AMC, CZ, CF, CTX, CAZ, TE, SXT, G, C	8	0.25	0.125	NT	NT
Gi-CC-28-T		*aac(6’)-Ib-cr*	-	AM, CZ, CF, TE, SXT	16	0.06	0.25	NT	NT
Gi-CC-35-T		*qnrB4*	-	AM, TE, C	8	0.125	0.06	NT	NT
Gi-CC-37-T		*qnrS1*	TEM-1	AM, CZ, FOX, G	4	0.125	0.25	NT	NT

a PMQR, plasmid-mediated quinolone resistance; NT, not tested

b AM, ampicillin; AMC, amoxicillin-clavulanic acid; CZ, cefazolin; CF, cephalothin; FOX, cefoxitin; CXM, cefuroxime; CAZ, ceftazidime; CTX, cefotaxime; C, chloramphenicol; G, gentamicin; SXT, sulfamethoxazole/trimethoprim; TE, tetracycline

c MICs, minimum inhibitory concentrations; NA, nalidixic acid; ENR, enrofloxacin; CIP, ciprofloxacin

d QRDR, quinolone-resistance determining region; WT, wild type
